# Hippocampal sclerosis dementia: an amnesic variant of frontotemporal degeneration

**DOI:** 10.1590/S1980-57642013DN70100013

**Published:** 2013

**Authors:** Chiadi U. Onyike, Olga Pletnikova, Kelly L. Sloane, Campbell Sullivan, Juan C. Troncoso, Peter V. Rabins

**Affiliations:** 1 Division of Geriatric Psychiatry and Neuropsychiatry, Department of Psychiatry and Behavioral Sciences, Johns Hopkins School of Medicine, Baltimore, USA.; 2 Division of Neuropathology, Department of Pathology, Johns Hopkins School of Medicine, Baltimore, USA.; 3 Division of Medical Psychology, Department of Psychiatry and Behavioral Sciences, Johns Hopkins School of Medicine, Baltimore, USA.; 4 Division of Neuropathology, Department of Pathology, Johns Hopkins School of Medicine, Baltimore, USA. Department of Neurology, Johns Hopkins School of Medicine, Baltimore, USA.

**Keywords:** dementia classification, hippocampal sclerosis, frontotemporal dementia, neuropsychiatry

## Abstract

**OBJECTIVE:**

To describe characteristics of hippocampal sclerosis dementia.

**METHODS:**

Convenience sample of Hippocampal sclerosis dementia (HSD) recruited from the Johns Hopkins University Brain Resource Center. Twenty-four cases with post-mortem pathological diagnosis of hippocampal sclerosis dementia were reviewed for clinical characterization.

**RESULTS:**

The cases showed atrophy and neuronal loss localized to the hippocampus, amygdala and entorrhinal cortex. The majority (79.2%) had amnesia at illness onset, and many (54.2%) showed abnormal conduct and psychiatric disorder. Nearly 42% presented with an amnesic state, and 37.5% presented with amnesia *plus* abnormal conduct and psychiatric disorder. All eventually developed a behavioral or psychiatric disorder. Disorientation, executive dysfunction, aphasia, agnosia and apraxia were uncommon at onset. Alzheimer disease (AD) was the initial clinical diagnosis in 89% and the final clinical diagnosis in 75%. Diagnosis of frontotemporal dementia (FTD) was uncommon (seen in 8%).

**CONCLUSION:**

HSD shows pathological characteristics of FTD and clinical features that mimic AD and overlap with FTD. The findings, placed in the context of earlier work, support the proposition that HSD belongs to the FTD family, where it may be identified as an amnesic variant.

## Abstract (pt)

**OBJETIVO:**

descrever as características da demência com esclerose hipocampal.

**MÉTODOS:**

Uma amostra de conveniência de HSD foi recrutada no *Johns Hopkins University Brain Resource Center*. Vinte e quatro casos com diagnóstico patológico pós-morte de demência com esclerose hipocampal foram revisados para caracterização clínica, utilizando variáveis obtidas de prontuários médicos.

**RESULTADOS:**

A maioria dos pacientes (79,2%) tinha amnésia no início, e a maioria (54,2%) apresentava comportamento anormal e transtorno psiquiátrico. Aproximadamente 42% apresentavam um estado amnésico e 37,5% amnésia mais conduta anormal e transtorno psiquiátrico. Todos acabaram por desenvolver um distúrbio comportamental ou psiquiátrico. Desorientação, disfunção executiva, afasia, agnosia e apraxia foram incomuns no início. A doença de Alzheimer (AD) foi o diagnóstico clínico inicial em 89% e o diagnóstico clínico final em 75%. A maior parte dos casos mostrou atrofia e perda neuronal localizada no hipocampo, amígadale cortex entorrinal.

**CONCLUSÃO:**

HSD apresenta características patológicas de DFT e as características clínicas que mimetizam AD. Os resultados, colocadas no contexto do trabalho anterior, suporta a ideia de que HSD pertence à família FTD, em que pode ser identificada como uma variante amnésica.

## INTRODUCTION

Clinical classifications of dementia organize distinctive phenotypic features that, in turn, reflect the nature and distribution of defining pathological characteristics. Likewise, pathological classifications organize distinctive morphological and histochemical features into disease types that vary in their phenotype. Hippocampal sclerosis has proved difficult to classify, owing to ambiguity in its clinical presentations and pathological characteristics. It is characterized, pathologically, by severe loss of pyramidal neurons and gliosis in the CA1 region and subiculum of the hippocampus. Frequently it is seen in concert with widespread neurodegeneration in Alzheimer disease (AD),^1^ wherein the pathological classification tends to be straightforward. It is seen frequently in frontotemporal dementia (FTD); it has been found in >75% of tau-negative FTD with ubiquitin-positive inclusions,^1-3^ and TAR-DNA binding protein 43 (TDP-43) inclusions are present in nearly 90% of dementia cases (AD and FTD) showing hippocampal sclerosis.^4^ Furthermore, FTD carrying the recently described^5,6^ chromosome 9 open reading frame 72 (C9ORF72) mutation shows hippocampal sclerosis alongside widespread cortical and subcortical pathology.^7,8^ HSD with tauopathy has also been reported,^9^ showing characteristics that fit the current neuropathological classification for FTD.^10,11^ Hippocampal sclerosis as part of widespread neuropathology has also been seen in other dementias,^12,13^ such as vascular dementia, dementia with Lewy bodies, progressive supranuclear palsy and corticobasal degeneration.

Isolated hippocampal sclerosis associated with dementia is not uncommon in large neuropathology series,^4,13-17^ but the features of a hippocampal sclerosis dementia (HSD) syndrome have not been settled. Thus the diagnosis is rarely made on clinical grounds. Nevertheless HSD is not uncommon in elders; it has been retrospectively identified in 12% of autopsy cases from a community series of dementia that fit clinical criteria for AD.^18^ Indeed it usually mimics the AD phenotype,^15,19,20^ but may also present clinical^21^ and neuropathological^22^ features suggestive of frontotemporal degeneration (FTD). The pathological and clinical data indicate a relationship, albeit ambiguous, between "pure" HSD and FTD. However it remains uncertain what the phenotype is and how the cases might be identified in clinics. If HSD is a *forme fruste* (or variant) of FTD, one may anticipate shared features. In this report we describe the clinical and neuropathological characteristics of consecutive HSD cases identified from the Johns Hopkins Brain Research Center, and ask whether presenting features are similar to those of FTD.

## METHODS

**Selection of cases.** We identified 25 cases in the Johns Hopkins University Brain Resource Center who had a pathological diagnosis of HSD. Subjects were selected from a database of brain autopsies performed by the Johns Hopkins Hospital Division of Neuropathology between 1985 and 2002. The cases were referred from clinical and research settings within and outside of Johns Hopkins. One case, referred from outside Johns Hopkins, was excluded because clinical data could not be found and pathology slides and tissue were not available. Demographic variables and clinical variables were abstracted from medical records. The Johns Hopkins IRB approved this study.

**Neuropathology.** We identified the gross pathologic features by examining brain slices fixed in 10% buffered formaldehyde. The original microscopy slides, which were all stained with hematoxylin-eosin (H&E) and Hirano silver, were examined. Tissue sections were immunostained for ubiquitin (DakoCytomation, polyclonal rabbit, 1:500), tau (monoclonal antibody diluted 1:50; Sigma-Aldrich, St. Louis, MO), phosphorylated Tau (PHF1, monoclonal, gift of Dr. Peter Davies, Albert Einstein College of Medicine, 1:100), TDP-43 (ProteinTech Group, polyclonal rabbit, 1:500), ubiquilin-2 (Novus, monoclonal 5F5, 1:500), and P62 (BD Transduction Laboratories, mouse anti-p62 lck ligand, 1:100). Immunoreactivity was visualized using diaminobenzidine (DAB) reaction.

**Data analysis.** Data was analyzed using Stata 11.^2^. We report descriptive statistics.

## RESULTS

**Clinical observations.** Twenty-four cases are described in the Table. The sample is Caucasian and 54.2% male. Median age at autopsy was 79 years, interquartile range 73.5-83. Duration of illness was 4.7-14.3 years. Most cases (79.2%) had amnesia at onset and 54.2% presented with behavioral disorders - apathy, neglect of self-care, perseveration, compulsion, disinhibition, restless and irritability. Features appearing in the first three years of illness defined the syndrome at illness onset. The presenting syndromes were: amnesic dementia in 41.7%, amnesia plus behavioral and psychiatric disorder in 37.5%, and non-amnesic behavioral dementia in 16.7%. One case presented with amnesia and a persistent delusional psychosis. Another 4.2% of cases had presentations defined by other cognitive features (such as executive dysfunction); a research subject who did not have dementia at enrollment was also included in this group. Disorientation, executive dysfunction, aphasia, agnosia and apraxia were uncommon at onset, and appeared later in all the cases. All cases eventually developed apathy, abnormal conduct, depression, labile emotions, irritability or agitation. About half the cases developed hyperphagia, the rest anorexia and hypophagia. In general, feeding disorders appeared later in the illness. Two patients showed early akinesia and parkinsonism, whereas in others motor features (primarily tremor and gait disorder) developed later. AD was the initial clinical diagnosis in 87.5%, and the final clinical diagnosis in 75%. Two patients had final diagnosis of FTD.

**Table 1:** Characteristics of hippocampal sclerosis dementia.

ID	Sex	Age*	Syndrome**	First dx***	Final dx***	Tau	TDP43	UBQ	FC	TC	HC	ERC
1	M	64	Amnesia	AD	HSD			P				
2	F	82	Amnesia	AD	AD		N	N	++	-	+++	+++
3	F	94	Amnesia	AD	AD	N	N	P	-	-	+++	+
4	M	55	Abnormal conduct	AD	AD	N	P	P	-	-	-	-
5	M	79	Amnesia	AD	AD		N		-	-	+++	+
6	M	69	Amnesia	AD	AD	N	P	P	+	++	+++	+++
7	F	74	Amnesia + abnormal conduct + irritability/agitation	AD	AD	N	P	P	++	++	+++	+++
8	M	64	Amnesia	AD	AD	N	N	P	-	-	+++	+
9	F	79	Amnesia + irritability/agitation	AD	FTD	N	P	P	++	++	+++	+++
10	F	79	Amnesia + abnormal conduct + parkinsonism	PSP	PSP		N		-	-	-	-
11	M	73	Amnesia + abnormal conduct + irritability/agitation	AD	AD	N	P	P	+	+	+++	+++
12	F	74	Amnesia + psychosis + irritability/agitation	AD	AD	N	N	N	+	+	+++	+
13	M	81	Amnesia + abnormal conduct + irritability/agitation	AD	FTD	N	P	P	+	+++	+++	+++
14	M	94	Amnesia	AD	AD	N	N	N	+	++	+++	++
15	M	55	Agnosia + abnormal conduct + irritability/agitation	DNOS	DNOS	N	P	P	-	-	+++	+
16	F	89	Normal	Normal	DNOS	N	P	P	-	-	-	-
17	F	82	Amnesia + aphasia + abnormal conduct + irritability/agitation	AD	AD	N	P	P	+	++	+++	+++
18	F	96	Amnesia + agnosia + apraxia	AD	AD	N	P	P	++	+++	+++	+++
19	M	79	Amnesia	AD	AD				-	-	+++	-
20	M	77	Executive dysfunction + abnormal conduct + parkinsonism	AD	AD	P	N	P	++	++	+++	+++
21	F	90	Amnesia + abnormal conduct	AD	AD	N	N	P	-	-	+++	+
22	F	83	Amnesia	AD	AD	N	N	P	-	+	+++	+++
23	M	82	Abnormal conduct + neglect of self-care + irritability/agitation	AD	AD	N	P	P	++	+++	+++	+++
24	M	83	Amnesia + neglect of self-care + irritability/agitation	AD	AD	N	P	P	-	-	+++	+++

Cases 1, 3-8, 12, 15 and 17-23 were referred by research projects; others referred by clinics.

* Age at death (years).

** Clinical syndrome at illness onset.

*** First and Final clinical diagnosis; AD: Alzheimer disease; FTD: frontotemporal dementia; HSD: hippocampal sclerosis dementia; normal: research control subject; PSP: progressive supranuclear palsy; DNOS: dementia not otherwise specified. Neuropathological characteristics described by immunohistochemical and neuroanatomical aspects: Tau; TDP-43: TAR DNA binding protein 43; UBQ: ubiquitin; FC: frontal cortex; TC = temporal cortex; HC = hippocampus; ERC: entorrhinal cortex. P: positive immunohistochemical staining for tau, TDP-43 and/or ubiquitin; N: negative. Plus (+) signs denote neuronal loss and atrophy (mild +, moderate ++, severe +++); dash (-) indicates minimal atrophy.

**Neuropathologic observations.** Pathological features are described in the Table. All cases showed atrophy and degenerative changes predominantly in hippocampus, amygdala and entorrhinal cortex, and relatively modest changes in the frontal and temporal cortices. In 10 patients (41.7%), neuronal loss and atrophy were localized to the hippocampus and entorrhinal cortex. About half of the patients showed caudate atrophy and nigral degeneration. Atrophy of cerebellum or brainstem was not observed in any patients.

Ubiquitin-positive, TDP-43 positive, tau-negative perikaryal inclusions were found in neurons of the hippocampus and cerebral cortex in 11 patients (45.8%). These cases showed p62 inclusions in the hippocampus; about half also showed ubiquilin-2 positive inclusions. Another 4 cases showed ubiquitin-positive, TDP-43 negative perikaryal inclusions. Tau-positive perikaryal inclusions were found in one case. None of the cases showed hippocampal or cortical amyloid deposition.

## DISCUSSION

HSD is difficult to classify clinically because of its predominantly amnesic presentation, which suggests the AD syndrome^23^ and makes it difficult to identify in the clinic. It has also been difficult to classify pathologically. As in most other reported series, our patients with HSD mimicked AD. However, many also had behavioral features consistent with FTD - abnormal conduct, neglect of self-care, perseveration and disinhibition.

An earlier study analyzed behavioral characteristics in 19 of these cases^21^ and showed high prevalence during the illness of behavioral features consistent with FTD. This study complements that one by showing that the initial presentation consists of clinical syndromes characterized by amnesia and, frequently, behavioral and psychiatric disorder. Thus, our findings conform with other descriptions of HSD as an amnesic progressive dementia that is similar to AD^12,19^ and occurrs mainly in elders,^4,13,14^ and also shows that initial presentations often include behavioral states that are characteristic of the FTD syndrome. This amnesic presentation was illustrated in a community-based study^18^ that found HSD on pathology examination in 12% of elders who had clinical diagnosis of AD. Unfortunately it is not yet possible to reliably discriminate HSD from AD in the clinic, despite the behavioral features - although there is preliminary evidence that HSD shows relative preservation of executive functions.^18,24^

From the perspective of a clinical evaluation, it would be valuable to understand if HSD shows anatomical changes detectable on brain MRI or FDG PET scanning. To our knowledge this has not been done. We do not have the brain imaging data for many of the cases, and what is available varies widely in quality, so we have not addressed that issue in this study. In our view it is reasonable to anticipate unilateral or bilateral hippocampal sclerosis on MRI scans, but it is uncertain what the predictive value of the finding would be (i.e., to what extent such a finding would distinguish HSD from early AD).

As noted earlier, hippocampal sclerosis is often a feature of widespread neuropathology in FTD; however, hippocampal neuronal loss and astrocytic gliosis have been found to be more focal and severe in HSD than in FTD.^2^ To the extent it constitutes a distinct pathologic or phenotypic entity, HSD expands the heterogeneity of FTD. Pathologic heterogeneity in FTD, first described 40 years ago,^25^ forms the basis for its phenotypic heterogeneity. The features of HSD are consistent with its pathological classification as a form of FTD, but its predominantly amnesic phenotype has not been accommodated in the clinical classification. We share the view that HSD may represent a distinct FTD phenotype;^26^ we propose that it be included as an amnesic variant of FTD in an updated clinical classification. We recognize the practical problem posed by its clinical similarity to AD, by the difficulty in distinguishing the two on clinical grounds, and by its comparative rarity.

The main limitation of our sample and analysis is the reliance on ascertainment from brain banks, which entails differences in the sources of cases and variability in the quality of the clinical data. This limitation is mitigated by the fact that most clinical and pathologic observations of HSD have been replicated. However, prospective ascertainment is difficult, because of the close clinical similarity between HSD and AD and the high costs of diagnostic expertise and pathological examinations. An alternative approach is retrospective analysis of data from dementia registries that do systematic ascertainment of symptoms and use standardized psychometric and brain imaging measurements, such as is (partially) embodied by the National Alzheimer's Coordinating Centers^27^ in the United States.

In summary, HSD shows pathological characteristics consistent with FTD and a clinical presentation of amnesia mimicking AD plus phenotypic overlap with FTD. Research findings, taken together, indicate that TDP-43 positive HSD belongs to the FTD family. It would appear that tau positive HSD also fits in this family, except where pathological features are consistent with AD or other neurodegenerative disorder. As the amnesic presentation makes HSD difficult to distinguish from AD, it is important to develop methods - brain imaging, genetic screening, or bioassays - to facilitate the clinical diagnosis. Prospective and registry studies can also provide opportunities for finer analyses of the phenotype.
